# Arthritis in Two Patients With Partial Recombination Activating Gene Deficiency

**DOI:** 10.3389/fped.2019.00235

**Published:** 2019-07-05

**Authors:** Kevin Y. Wu, Pooja Purswani, Boglarka Ujhazi, Krisztian Csomos, Mihailova Snezhina, Naumova Elissaveta, Stefan Stefanov, Svetlana Sharapova, Maryssa Ellison, Diana Milojevic, Sinisa Savic, Ravishankar Sargur, Jolan E. Walter

**Affiliations:** ^1^Division of Pediatric Allergy and Immunology, Department of Pediatrics, University of South Florida, St. Petersburg, FL, United States; ^2^Johns Hopkins All Children's Hospital Children's Research Institute, St. Petersburg, FL, United States; ^3^Department of Pediatrics, University of South Florida, St. Petersburg, FL, United States; ^4^Department of Clinical Immunology, University Hospital Alexandrovska, Medical University, Sofia, Bulgaria; ^5^Clinic of Rheumatology, Cardiology and Hematology, University Pediatric Hospital, Medical University, Sofia, Bulgaria; ^6^Belarusian Research Center for Pediatric Oncology, Minsk, Belarus; ^7^Division of Rheumatology, Department of Medicine, Johns Hopkins All Children's Hospital, St. Petersburg, FL, United States; ^8^Department of Clinical Immunology and Allergy, Leeds Institute of Rheumatic and Musculoskeletal Medicine, St. James's University Hospital, Leeds, United Kingdom; ^9^Sheffield Teaching Hospitals Foundation NHS Trust, Leeds, United Kingdom

**Keywords:** recombination activating gene, RAG deficiency, rheumatoid arthritis, primary immunodeficiency, tocilizumab, refractory arthritis

## Abstract

Autoimmunity is becoming an increasingly recognized complication in patients with primary immunodeficiencies (PIDs), including a variety of combined immune deficiencies such as Recombination Activating Gene (RAG) defects. The approach to treating autoimmunity in PID patients is complex, requiring a balance between immunosuppression and susceptibility to infection. Inflammatory arthritis is a feature of immune dysregulation in many PIDs, and the optimal treatment may differ from first line therapies that usually consist of disease-modifying anti rheumatic drugs (DMARDs). An example of mechanism-based therapy of arthritis in PID uses blockade of IL-6 signaling with tocilizumab for patients with STAT 3 gain-of-function (GOF) mutation and augmented IL-6 pathway. Herein, we describe two PID cases with arthritis who were found to have defects in RAG. One patient with refractory inflammatory arthritis experienced remarkable improvement in symptoms with tocilizumab therapy. Arthritis can be a clinical feature of immune dysregulation in RAG deficiency, and tocilizumab therapy has been suggested to have utility in treatment of arthritis in RAG deficiency.

## Introduction

The co-occurrence of autoimmunity and immunodeficiency was previously thought to be paradoxical, however, recent understanding of immunology has shed light on common immune dysregulating mechanisms in both. As these complicated mechanisms are still being elucidated, treatment strategies for autoimmune conditions are not well-defined in this phenotype. One of the best studied treatment regiments for autoimmunity is that of rheumatoid arthritis (RA) in adults and juvenile idiopathic arthritis (JIA) in children. The initial months of RA are critical for initiation and escalation of therapies as using methotrexate for standard, first-line monotherapy is sufficient for only one-third of RA patients. For second and third line therapies, the American College of Rheumatology provides a decision tree that lists a plethora of biologics for treating RA and JIA with various efficacies and adverse event profiles, making selection of the right medication for specific patients difficult (1, 2). Immune mechanism-based selection is uncommon in treatment of arthritis in the general population. However, there is a population of patients with PIDs where it is imperative to balance the benefits of immunomodulating therapies with the potential of increasing susceptibility to infection. By determining the immune mechanism underlying the patient's disease, we will be able to determine personalized targeted therapy.

Inflammatory arthritis in PIDs arises via heterogenous mechanisms involving immune dysregulation in different lymphocyte subpopulations. PIDs defined by B-cell pathology are linked to arthritis: in X-linked agammaglobulinemia (XLA), 7% of patients had a diagnosis of arthritis, and in common variable immune deficiency (CVID), 3.2% of patients were diagnosed with inflammatory arthritis (3, 4). Regulatory T-cell (Treg) pathology is also linked to arthritis in 3 major cohorts, including patients with LRBA (31 pts), CTLA4 (133 pts) deficiency, and STAT3 GOF mutations (13 pts), in which 26, 3, and 26% of patients had arthritis, respectively (5–7). Inflammatory arthritis is also associated with other combined immune deficiency (CID) syndromes. In 55 patients with Wiskott Aldrich syndrome, 29% experienced arthritis as a complication (8). Arthritis has also been a known phenomenon in ZAP-70 deficient mouse models of CID (9). Finally, an association between RAG, the major player in VDJ recombination, and arthritis is emerging (10). Patients with RAG and combined immune deficiency associated with granulomas and/or autoimmunity (CID-G/A) may present with immune dysregulation in all ages (11). Chronic refractory arthritis was part of clinical presentation in a systemic lupus erythematosus patient with a heterozygous pathogenic RAG mutation and low RAG function measured by reduced receptor editing (12). To examine the association between RAG deficiency and arthritis, we report two RAG-deficient patients with CID that may lay precedent for further investigation.

## Case Report A

**Figure 1:**
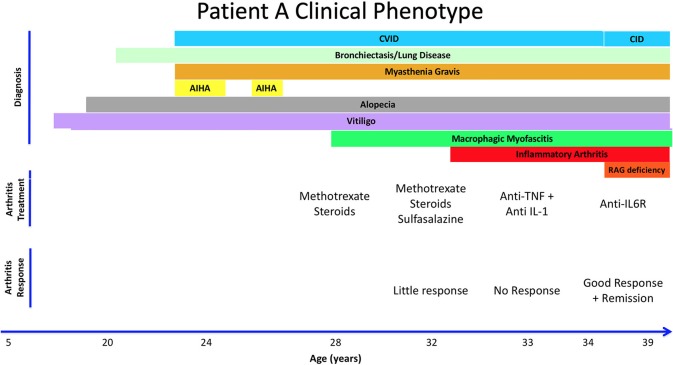
Summary of Patient A's clinical history, treatment, and response to therapy. Autoimmune Hemolytic Anemia (AIHA). Combined Immune Deficiency (CID). Common Variable Immunodeficiency (CVID). Hydroxychloroquine (HCQ).

Patient A is a 39-year old female from the United Kingdom who was diagnosed with probable CVID at age 24 after presenting with recurrent childhood infections, including pneumonias. She also experienced multiple autoimmune complications, including vitiligo in her adolescent years and developed alopecia at age 20 (Figure 1). At age 24, she was hospitalized with severe bulbar palsy and was diagnosed with myasthenia gravis without thymoma. At this time, immunological studies were performed, which were positive for lymphopenia, neutropenia, autoimmune hemolytic anemia (AIHA), and a IgG deficiency (3 g/L), but without deficiency in IgM or IgA. Because of lack of response to two challenges of pneumococcal vaccines, deficiency in one immunoglobulin isotype, and negative test for hyper IgM syndrome, a diagnosis of probable CVID was made (Table 1). She was subsequently started on IVIG replacement, and azathioprine and pyridostigmine for her myasthenia gravis. At age 28, she presented with restriction in movement of joints, prompting synovial fluid analysis which was negative for inflammatory cells. Subsequently, a muscle biopsy was performed revealing macrophagic myofasciitis. She initially responded well to treatment with steroids, but experienced 2 further relapses of macrophagic myofasciitis over 4 years, requiring steroids. Methotrexate was started as a steroid-sparing agent and azathioprine was stopped. She then developed knee arthritis, and another synovial fluid analysis was done on her right knee, which demonstrated inflammatory cells. She was diagnosed with inflammatory arthritis at age 33. She continued on methotrexate and steroids with addition of sulfasalazine, but due to suboptimal response, sulfasalazine and methotrexate were replaced with anti-TNFα with no response, then with anti-IL-1 (Anakinra) with no significant improvement. At age 34, patient A visited a rheumatology clinic and was started on tocilizumab, an anti-IL-6 biological agent, resulting in complete remission of joint-related symptoms. She has been in remission for the past 5 years. As part of the 2018 BRIDGE study on genetic defects in PID cohorts, she underwent whole exome sequencing and was found to have a compound heterozygous RAG2 mutation (c.a. G1352C, b. T629C; p.a. I210T, b. G451A), causing an average recombination activity of 6.4 ± 0.2% (11). Elevated IgA reflects inflammation and patient had progressive B cell loss and very low fraction of naïve T cells. Anti-cytokine antibodies were also present (Table 1). Together, based on the immunological phenotype and reduced recombination activity, CID phenotype was established.

**Table 1:** Immunological phenotype for Patient A and B.

**Immune Phenotype**	**Units**	**Patient A**	**Immune Phenotype**	**Units**	**Patient B**
**RAG Mutation**		**RAG2**	**RAG Mutation**		**RAG1**
		**Allele a (c.G1352C p.I210T)**			**Allele a and b**
		**Allele b (c.T629C p.G451)**			**(c. C1443T p. A444V)**
Relative Recombination Activity^*^	%	6.4 ± 2	Relative Recombination Activity^*^	%	1.4 ± 0.2
CD3+	Cell/μl	101–667	CD3+	Cell/μl	84–697
CD3+	%Ly	56–66.8	CD3+	%Ly	19–45
CD4+	Cell/μl	54–290	CD4+	Cell/μl	62–254
CD4+	%Ly	43–53.8	CD4+	%Ly	11–19
Naive CD4+CD45RA+	%Ly	5	Naive CD4+CD45RA+	%Ly	3.6–5.8
CD4+CD45RO+	%Ly	95	CD4+CD45RO+	%Ly	94.2
CD4+ CD25+ CD127low	% Ly	4.2	Treg CD4+CD25+CD127low	%CD4+	4.0
CD8+	Cell/μl	30–180	CD8+	Cell/μl	18–302
CD8+	%Ly	11.3–17	CD8+	%Ly	4–25
CD56+	Cell/μl	150–244	CD56+	Cell/μl	580
CD56+	%Ly	28.1–36	CD56+	%Ly	50–62
CD19+	Cell/μl	10–50	CD19+	Cell/μl	6
CD19+	%Ly	3.6–7	CD19+	%Ly	0.5 – 9
IgG	g/l	467^**^	IgG	g/l	1027–1431^**^
IgA	g/l	930	IgA	g/l	76.7–103.9
IgM	g/l	234	IgM	g/l	46.6–77.6
anti–IFNα		Yes	Anti–IFNα		N/A
anti–IFNω		No	Anti–IFNω		N/A
anti–IL−12		Yes	Anti–IL−12		N/A
IL−6	pg/ml	22.4 (0–15)	IL−6	pg/ml	0 (0–15)
**Rheumatological Phenotype**	**Units**	**Patient A**	**Rheumatological Phenotype**	**Units**	**Patient B**
Rheumatoid Factor	IU/ml	16 (0–15)	TRECs during NBS		0
Anti AchR	nmol/L	>100nmol/L	Anti-dsDNA	U/ml	11.4– 37.2 (<20)
		positive	APA IgM	U/ml	15.3 (<10)
Anti-Skeletal Muscle	IU/ml	13 (0-55)	B2-glycoprotein	U/ml	55.7 (<5)
Anti-dsDNA	U/ml	<1	ANA		(1:1280)
Anti-CCPAb		negative	ESR		Elevated
ANA		negative	C-Reactive Protein		Elevated
ENA screen			Complement C3	g/l	1.324 (0.75–1.65)
Anti-Skeletal Muscle			Complement C4	g/l	0.137 (0.20–.065)
**Arthritis**		**Inflammatory**	**Arthritis**		**Juvenille idiopathic**
Symmetry		Symmetric	Symmetry		Asymmetric
Joints Involved		Knees; no small joints	Joints Involved		Multiple joints
Erosive		Non-erosive	Erosive		Non-erosive

Values are provided as a range, collected over the course of patient's clinical history.

* Recombination Activity represents values previously reported (11)

** *IgG level was recorded pre-IVIG therapy*.

## Case Report B

**Figure 2:**
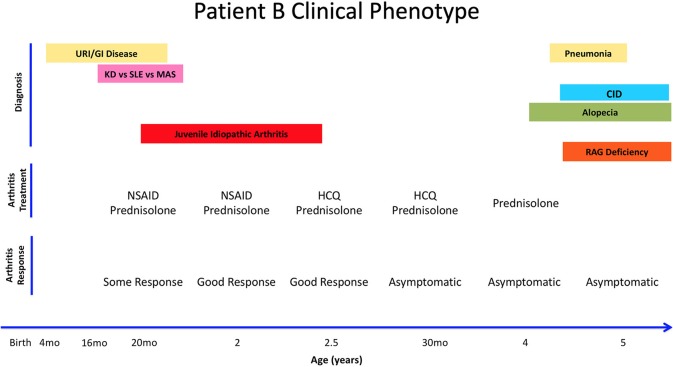
Summary of Patient B's clinical history, arthritis treatment, and response to therapy. Anti-Nuclear Antigens (ANA). Combined Immune Deficiency (CID). Kawasaki's Disease (KD). Macrophagic Activation Syndrome (MAS). Non-Steroid Anti-Inflammatory Drug (NSAID). Systemic Lupus Erythematosus (SLE). Upper Respiratory Infections (URI).

Patient B is a 5 year-old female from Bulgaria who was born full-term with no complications, but had multiple gastrointestinal and upper respiratory infections starting at 4 months of age (Figure 2). She required hospitalization at 14 months for bacterial gastroenteritis after presenting with high fever, diarrhea, decreased oral intake, and was subsequently treated with antibiotics. Two months later, she was hospitalized again with fever, rash, swelling of the legs, feet, and fingers, with concern for Kawasaki's disease after live MMR vaccination. Eventually, she was diagnosed with macrophage activation syndrome (MAS), successfully treated with parenteral and oral corticosteroids with good effect. Given that she was previously hospitalized with a bacterial infection and recurrent infections since 4 months of age, she also had a workup for immunodeficiency, which raised a suspicion for primary immune deficiency (Table 1).

At 20 months of age, she presented with another episode of fever and swelling of multiple joints with laboratory workup revealing leukopenia, elevated IgM, elevated CRP, elevated ESR, elevated ANA titer (1:1,280), and positive anti-dsDNA and anti-phospholipid IgM antibodies. At this time, she was diagnosed with inflammatory arthritis, with differential diagnosis of systemic lupus erythematosus as she fulfilled the WHO classification criteria for SLE (4/11 criteria: arthritis, leucopenia, positive ANA and positive dsDNA and APL). Her joint symptoms were controlled with NSAIDs and glucocorticoids. At 30 months of age, elevated ANA titers prompted therapy with hydroxychloroquine and glucocorticoids that resulted in a reduction in ANA titer to 1:320. Patient B was asymptomatic until 4 years of age, when she developed alopecia that was thought to be a side effect of hydroxychloroquine and therefore discontinued. Approximately 8 months later, she was hospitalized with pneumonia.

Because of her clinical history and abnormal immune phenotype, patient B's was further investigated. Fortunately, cord blood was obtained at birth and preserved at the National Public Cord Blood Bank. Immunological testing of the cord blood revealed an absence of T cells and B cells, and T-cell receptor excision circles (TREC) were undetectable. This led her clinicians to search for SCID-related genetic defects, revealing a homozygous *RAG1* mutation (c. C1443T, p. A444V) which causes a recombination activity of 1.4 ± 0.2% (13). Patient B had low naïve CD4 count, declining B cell numbers, and expansion of the NK cells. Although patient B had hypergammaglobunemia, she had a broad autoantibody profile (Table 1). Together, based on the immunological phenotype and reduced recombination activity, CID phenotype was established.

## Discussion

Patient A and patient B have immunodeficiency, autoimmunity, and hyperinflammation attributable to RAG deficiency. Patient A was classified as “probable CVID,” based on her hypogammaglobulinemia and lack of response to pneumococcal vaccination. However, she displays a phenotype characteristic of combined immunodeficiency with granulomas or autoimmunity (CID-G-AI). In contrast, Patient B has hypergammaglobulinemia, but nevertheless displays a CID phenotype based on absent TRECs, low naïve CD4 T cell count and low B cell count (Table 1). Both patients share a history of arthritis and alopecia, with patient A having additional complications including cytopenia, myasthenia gravis, and vitiligo. In CID-G-AI, autoimmune conditions tend to worsen later in life especially after exposure to environmental triggers such as viral challenge; this phenomenon may have led to Kawasaki-like symptoms in patient B after MMR vaccination. Along this vein, possible exacerbation of Patient B's currently moderate symptoms into more severe, treatment-resistant autoimmune conditions, such as refractory arthritis experienced by Patient A, is a major concern.

Ultimately, the goal in treating immunodeficient patients is to perform hematopoietic stem cell transplantation (HSCT) for immune reconstitution, essentially curing the defect in patient immune cells. Drugs to treat autoimmune complications often serve as a bridging therapy to keep the patient as healthy as possible until eventual transplantation. Our two patients exemplify the complicated scenarios PID patients encounter when deciding on which immunomodulating therapies to treat autoimmune complications. Patient A displayed suboptimal responses to first line DMARD therapies for arthritis, requiring a targeted therapy, tocilizumab, to contain her arthritis. Patient B currently has a milder form of disease and did not require additional treatment after a short course of therapy. If Patient B's disease state worsens, particularly upon development of inflammatory arthritis that is refractory to first-line therapy, tocilizumab may be recommended based on her immune background.

Tocilizumab has been FDA approved for treatment of chronic inflammatory arthritis in adults (rheumatoid arthritis) and children (polyarticular juvenile idiopathic arthritis) who fail classical (synthetic) DMARDs and as first-line biological therapy for children with systemic subtype of juvenile idiopathic arthritis, a highly inflammatory disease characterized by high IL-6 blood levels. Tocilizumab monotherapy was shown to outperform methotrexate (MTX) as monotherapy in classical DMARD-naïve RA patients (14, 15). In a network meta-analysis studying adverse effects of biologics, all biologics including tocilizumab were associated with significantly higher adverse effects than MTX, including increased risk of serious infections. Tocilizumab conferred equal or less risk for infections when compared to other biologics such as TNF inhibitors, IL-1 antagonists, or other mAbs such as rituximab (16). Therefore, among biologicals, tocilizumab may be a safer and more powerful option for PID patients who are required to escalate therapy to address refractory inflammatory arthritis. Its use could be further justified in patients with PIDs with IL-6 overactivation.

IL-6 overactivation has been previously demonstrated in the context of five patients with immune dysregulation and STAT3 gain of function. Consequently, one patient in this cohort with refractory polyarthritis responded to IL-6 blockade with tocilizumab (17). As a parallel, partial RAG deficiency with immune dysregulation may result in treatment refractory autoimmune disorders such as chronic arthritis. In fact, an SLE patient with heterozygous RAG mutation and low receptor editing was reported with refractory arthritis (12). Other mechanisms of immune dysregulation in RAG deficiency also overlap with arthritis pathogenesis (18, 19). Abnormal Treg function is a classic finding in autoimmune conditions, and an abnormal Treg repertoire has been found both in patients with arthritis and in patients with RAG deficiency. Unlike in patients with STAT-3 GOF mutation clearly linked to IL-6 pathway overactivation, where IL-6 blockade appears to be a logical treatment option, choice of treatment in RAG deficiency is less clear. Although long term effects of IL-6 blockade in RAG-deficient patients is not yet known, the observation that refractory inflammatory arthritis in one of these patients was successfully treated with IL-6 blockade suggests tocilizumab as a treatment option. In addition, it creates a need to better define the role of IL-6 pathway in RAG deficiency.

Clinicians taking care of patients with chronic arthritis should consider exploring for underlying immune deficiency, such as RAG deficiency, in patients with atypical clinical presentation, even in the absence of the history of infections. For example, arthritis in systemic lupus erythematosus is typically non-erosive and although chronic, usually responds to treatment (steroids, methotrexate, hydroxychloroquine, etc). Hence, treatment resistant or erosive arthritis in SLE should raise a suspicion of underlying immune deficiency. JIA and RA are not typically associated with specific ANA antibodies (such as anti-dsDNA, RNP, SSA, SSB etc.). Hence, the presence of antibodies to specific nuclear antigens in a young child with chronic inflammatory arthritis should raise a suspicion and further workup for underlying immune deficiency.

## Concluding Remarks

Two new cases of partial RAG deficiency with multiple autoimmune complications including arthritis are reported, one with refractory arthritis successfully treated with anti-IL6 biological agent, tocilizumab. These two cases exemplify the broad clinical diversity of RAG deficiency, and also serve to increase awareness of PID background among patients with refractory arthritis. Better understanding of mechanisms of immune dysregulation in chronic arthritis will allow targeted therapies like tocilizumab to be selectively pursued, especially among vulnerable patients with PID.

## Informed Consent

Written, informed consent was obtained from patient A and from the father of patient B for publication of data contained in this case report.

## Author Contributions

KW drafted the initial manuscript, reviewed, and revised the manuscript. JW conceptualized and PP, DM, and JW assisted revision of the manuscript. BU and KC generated laboratory data in Tables 1, 2. RS, SiS, MS, ME, NE, SvS, and StS contributed sample acquisition, patient data, and reviewed the manuscript.

### Conflict of Interest Statement

The authors declare that the research was conducted in the absence of any commercial or financial relationships that could be construed as a potential conflict of interest. The reviewer YL declared a past co-authorship with one of the authors JW to the handling Editor.
